# A Plea for Conservative Minor Gynecology

**Published:** 1891-07

**Authors:** R. R. Kime

**Affiliations:** Atlanta, Ga.


THE ATLANTA MEDICAL AND SURGICAL JOURNAL.

VOL. VIII. JULY, 1891. No. 5.

TO CONTRIBUTORS.

Articles for publication must reach this office not later than 15th inst.; and are expected to be published exclusively in this JOURNAL. Extra copies of JOURNAL furnished contributors when requested. Reprints by special arrangement. Publication of an article does not necessarily imply endorsement of the views therein expressed.

MEDICAL AND SURGICAL JOURNAL, Post-office box 431.

### Original Communications.

### A PLEA FOR CONSERVATIVE MINOR GYNECOLOGY.

BY R. R. KIME, M. D., ATLANTA, GA.

[*Continued.*]

In all operative procedures the vitality of patient and the power to withstand shock and prevent infection should be considered. Has the patient sufficient vitality to carry on the reparative process after surgical procedure?

Has the patient sufficient physical and nerve force to withstand the shock of the operation? Can the operation be performed without infection? If these questions can be answered in the affirmative, operations of the greatest magnitude can be performed with almost absolute certainty of success. The amount of cutting, scraping and separating is not so much a consideration as the environments of patient and the manner in which the work is done. Especially is this true in gynecology.

Take a patient with a blood dyscrasia, lowered vitality, nervous system a wreck, with septic influences present, then the most trivial operation is likely to prove disastrous, even the introduction of the sound so much condemned of late by some writers.

Because a urethral sound is introduced into the male urethra and carries the gonococci backward to new fields of infection with serious results, or unskilfully used makes a false passage, shall it be condemned and excluded from the armamentarium of the genito-urinary surgeon? Neither should the uterine sound be abandoned because it carries infection or perforates the uterus when improperly used.

If vaginal canal, cervix or sound be unclean, the instrument introduced and infection follows, is the much abused passive instrument to blame or the introducer?

With clean vaginal canal, cervix and sound, as well as hands of the operator, no trouble is likely to follow dexterous use of the sound. By reference to the writings of several prominent gynecologists we find all give the stereotyped rule of introducing sound guided by the finger without use of the speculum, some even condeming *use of speculum*. They fail to mention in that connection the necessity of disinfecting vaginal canal or cervix. In fact I have seen some of the most eminent men in New York so use the sound, urging the necessity of disinfecting finger and sound but not a word as to the dangers lurking in the genital passages of patient waiting to be conveyed to new fields of infection. Let us consider such a procedure, sanctioned by all authorities so far as I know.

Suppose patient requiring examination is suffering with some disease of vulva, vagina, cervix, even the endometrium, from any of which there may be an irritating discharge retained in the vagina, forming a very suitable nidus for the development of germs, especially in so fertile a soil and condition as the vagina affords, through which introduce sound and what is likely to occur, especially if the endometrium be wounded.

Again suppose patient suffering with a specific vaginitis of a mild type or a severer form partially controlled by treatment in which the gonococci failed to gain entrance to that chamber where more important personages are developed, yet allowed the privilege of secreting itself between the rugæ of the vaginal mucosa, in the mucous membrane itself, or glands of Bartholini, there to propagate its species and renew its attacks, or even allowed the menial privilege so congenial to its nature of taking up its abode in some nook or cranny of the urethral canal evading a watery grave but not so securely located but that it may be dislodged by the examining finger and carried by the sound to the uterine cavity.

Even the ptomaines, the alkaloidal developments of the gonococci, to which add the heat, moisture, often the discharge of diseased conditions of vagina and adnexia, constitute a most active poison only awaiting the assistance of the unwary gynecologist to convey it to the uterine cavity to make its stately steppings felt. Another fruitful source of danger is the walking gonococci generator who, the night before the examination, playing the part of a lover, seeks pleasure in what should be sacred to the hymeneal altar and deposits not only the germs of a physiological process but of a diseased process as well, to wreak its vengeance on the unsuspecting female, or the husband who, recreant to his matrimonial vows, sought pleasure in forsaking virtue's paths and thus infects his innocent wife, to which the unwitting gynecologist acts the part of a conspirator and conveys the gonococci to the uterine cavity by the use of a *disinfected sound and finger.*

These are not fanciful pictures of dangers that never occur, but are true to nature and nature's laws violated.

Then, in view of these facts, shall the sound be used as directed in our text-books, often carrying germs and poisonous substances to the uterine cavity, producing serious lesions, perhaps death, when we have at command means to prevent such results? It should be a law inviolate never to introduce a uterine sound without first *disinfecting vaginal canal and cervix*, as well as sound, and examining finger, at least rendering them aseptic—clean. I would advise the following rules for introducing sound.

1. Exclude acute active pelvic inflammation and pregnancy.

2. Disinfect, or render aseptic, hands, instruments, vagina, cervix, and uterine cavity if it contain any deleterious substances.

3. Use Sims' or bivalve speculum, exposing cervix. *If proper disinfection, may discard speculum.*

4. Hold sound between thumb and first or second finger, dipping uterine end into pure carbolic acid or tincture iodine; cleanse it with absorbent cotton and introduce without force. Thus used, there is no danger, and all information the sound need give can be obtained. The question might be asked, what is the legitimate use of the sound?

I would say, to measure cavity oftener, confirm diagnosis of tumors, position of uterus, condition of internal os and corporeal endometrium.

The expert may diagnose size and position of uterus, presence of tumors, etc., by touch; the non-expert needs the evidence of the sound to confirm his diagnosis. The expert can introduce sound by *tactus eruditus without* use of the speculum causing infection and trouble; the non-expert, using speculum, disinfecting, using sound by sight and touch, will not cause trouble if he remembers not to use force and that the sound is not a uterine repositor.

To meet objections to use of speculum, I would reply that where displacement of uterus is sufficient to be classed as pathological so that it can be usually detected by examining finger, that examining finger, in making a proper examination, is as likely to displace uterus as the use of the speculum; that the sound is more often needed where the uterus is not so easily displaced, when tumors are present, and more especially to measure depth of uterus, ascertain condition of internal os and corporeal endometrium, which can be as readly obtained through a bivalve or Sims' speculum, especially by the non-expert, and with far greater safety to patient.

To briefly continue the subject of minor operative work I would say, in all minor gynecological work, exclude pregnancy, acute pelvic inflammations and pus accumulations, except where uterus contains putrid material, and give preparatory treatment; then, with aseptic operator, aseptic instruments and aseptic patient, little need be feared unless undue traction is made on uterine supports or adhesions from previous inflammatory deposits. Given a patient with lacerated cervix, lips everted, eroded, subinvolution, leucorrhœa, with reflex nervous manifestations, with consequent pelvic symptoms, prepare patient by constitutional and local treatment, operate.

If a young lady, generative organs not fully developed as a result of imperfect physical development, *severe pains during flow*, excessive nervous manifestations, weak, debilitated, and if not relieved by constitutional treatment, tonics, good food, out-door exercise, etc., dilate cervix, curette, introduce cervical drainage stem, keeping up use of stem for some time with judicious use of Faradic electricity. If she be married, such procedure will favor pregnancy, thereby often preventing a life of suffering. Certainly I do not claim this procedure will give relief where ovaries and tubes are at fault primarily.

If clinical history of case demonstrates dysmenorrhœa, deranged flow, "pain on passing sound through internal os, followed by slight hemorrhage, dilate, curette, drain, relieving patient."—(*Wyle.*)

If fungous degeneration of endometrium, "endometritis hyperplastica," metrorrhagia, with consequent weakening of vital forces not relieved by ordinary treatment, dilate, curette, drain.

In gonorrhœa the treatment should be active, energetic, certain. Properly dreaded by man, but should be far more so by woman. "Syphilis has slain its thousands; gonorrhœa its tens of thousands." It is only of late that the evil influences of gonorrhœa in the female are being recognized. The length of this paper forbids going into details, yet I would urge the necessity of destroying the gonococci, while yet in vaginal canal or urethra; failing of this, reach it in uterine cavity, if possible, by rapid dilatation of cervix, irrigation with mercuric chloride; then curette with dull curette to remove coating of albuminate of mercury and *dislodge and squeeze out* the burrowed gonococci, again irrigate with mercuric chloride, then apply carbolic acid or tr. iodine, afterward filling uterine cavity loosely with a ribbon of iodoform gauze, end left protruding from cervix, by which to remove it at expiration of twenty-four or forty-eight hours, at the same time giving appropriate treatment for condition of vaginal canal. Repeat irrigations and applications to uterine cavity every one or two days until disease is under control, thus preventing in many cases salpingitis, ovaritis, and pelvic peritonitis with pus formation, demanding abdominal section menacing life of patient.

The results obtained by these minor gynecological procedures with those presented in former article (ATLANTA MEDICAL AND SURGICAL JOURNAL, June, 1891), certainly demonstrate a sufficient plea for the continuance of conservative minor gynecology.

Conservative, because they relieve suffering woman of many of the ills common to her sex, preventing many pelvic complications which render the patient a sufferer for life or a fit subject for the abdominal surgeon.

Conservative, because such procedures often save the life of a wife, a mother, the anchor to a happy home.

